# A possible later stone age painting of a dicynodont (Synapsida) from the South African Karoo

**DOI:** 10.1371/journal.pone.0309908

**Published:** 2024-09-18

**Authors:** Julien Benoit

**Affiliations:** Evolutionary Studies Institute and School of Geosciences, University of the Witwatersrand, Johannesburg, South Africa; University of Silesia, POLAND

## Abstract

The Horned Serpent panel at La Belle France (Free State Province, South Africa) was painted by the San at least two hundred years ago. It pictures, among many other elements, a tusked animal with a head that resembles that of a dicynodont, the fossils of which are abundant and conspicuous in the Karoo Basin. This picture also seemingly relates to a local San myth about large animals that once roamed southern Africa and are now extinct. This suggests the existence of a San geomyth about dicynodonts. Here, the La Belle France site has been visited, the existence of the painted tusked animal is confirmed, and the presence of tetrapod fossils in its immediate vicinity is supported. Altogether, they suggest a case of indigenous palaeontology. The painting is dated between 1821 and 1835, or older, making it at least ten years older than the formal scientific description of the first dicynodont, *Dicynodon lacerticeps*, in 1845. The painting of a dicynodont by the San would also suggest that they integrated (at least some) fossils into their belief system.

## Introduction

A crucial, yet outstanding question about the history of palaeosciences is that of palaeontological indigenous knowledge in the South African Karoo. The almost continuous Permo-Jurassic fossil record of the South African Karoo chronicles the rise, diversification, and fall of the Therapsida, as well as the evolutionary origins of mammals, turtles, dinosaurs, and lizards, providing many intermediate species each documented by dozens of specimens [1–4]. The abundance of tetrapod fossils in the Karoo and their quality of preservation both make it a konzentrat and konservat lagerstatten [1,5,6]. Given the wealth of fossils that the Main Karoo Basin and other Karoo-aged basins have delivered, and the long human occupation of this part of the African continent, the existence of a long-standing indigenous knowledge of fossils is very likely [7,8]. Yet, the study of African indigenous palaeontology is still fairly young [7,9–12], and the evidence remains accordingly sparse and debatable, especially given the scarcity of written accounts. Although patchy, a growing record of geomyths, place names, written accounts, and archaeological evidence supports that many southern African cultures knew and, in some cases, inquired about the fossils around them [7,8].

**Fig 1:**
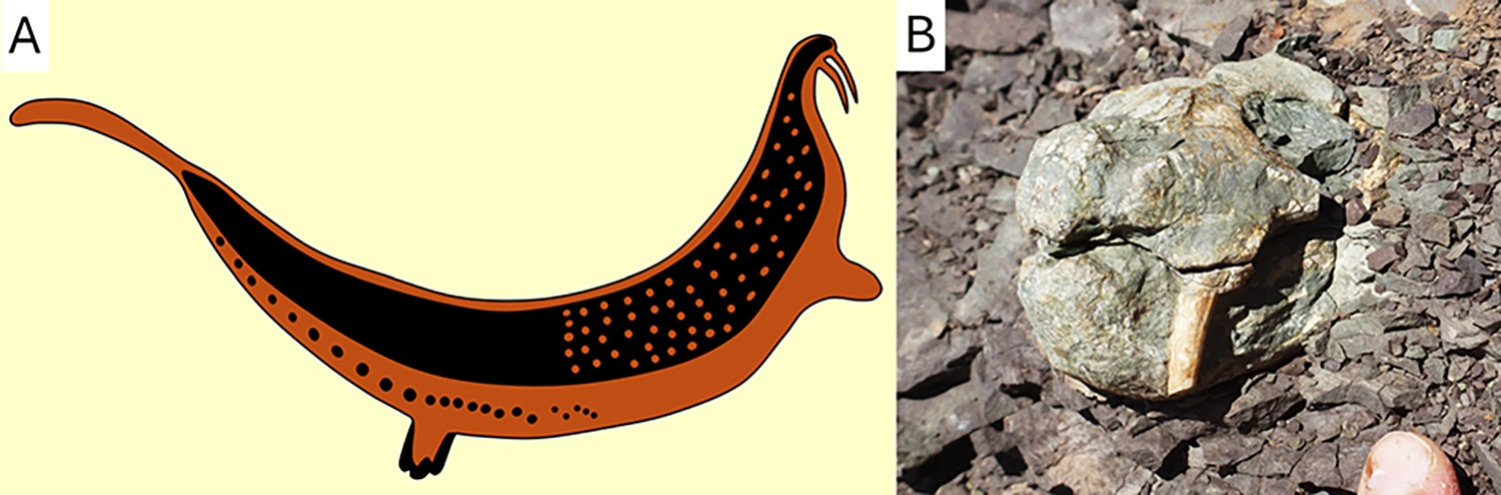
The tusked animal of the Horned Serpent panel compared to the skull of a dicynodont. A, the tusked animal of the Horned Serpent panel redrawn from Stow and Bleek [13]. B, skull of a *Diictodon feliceps* (BP/1/8140, Jasfontein, Victoria West District) photographed by the author in situ at the moment of its discovery, before excavation, and unprepared.

The Horned Serpent panel, in the Koesberg mountains, gorgeously depicts elements of the San culture and was partly figured and described by Stow and Bleek [13] in plates 36–39. The rock art was made at an unknown date by the “Bushmen of the East” [14], now referred to as the /Xam speaking San [15]. One of these elements is an unidentified animal that bears two enlarged tusks, making it superficially look like a walrus (Fig 1A). This walrus-like figure has been the object of much speculation regarding its identity, because no representative of the Odobenidae lives or has ever lived nearby sub-saharan African coasts [16–18]. Tusked creatures (more or less imaginary and composite) are not rare in San rock art, including tusked lion, snake, antelopes, and people; but in those cases, the tusks are always curved upwards, like they do in warthogs and bushpigs [19–21], not downwards as in the tusked animal from La Belle France (Fig 1A).

The question of the identity of this animal has been dismissed because it is seemingly a rain-animal from the San “spirit-realm” [13], which is spiritual by essence and does not have to be realistic [18]; however, this does not address the question of what inspired this figure in the first place. Even the most fantastic elements of San art, such as therianthropes and rain-animals, are based on actual animals and phenomena [13,21–25]. The San spiritual pantheon is directly inspired from their real-life environment and the fantastic beings they painted are thus always an amalgamation of different existing animals [13,14,21–23,25–27]. As such, whether fantastic or not, the question of what inspired this tusked animal remains open.

The /Xam speaking San, who made the Horned Serpent painting, occupied the Karoo area [15], a landscape in which the fossil-richness is mostly due to the overly abundant and often well-preserved dicynodonts, a group of tusked therapsids [1,2,5,28,29]. In many cases, their skulls are naturally exposed by erosion in spectacular ways (Fig 1B), making them easy to find and collect, and their tusks are so conspicuous that their anatomy is not difficult to interpret, even to the untrained eyes. The downturned tusks of dicynodonts resemble those of the tusked animal of the Horned Serpent Panel (Fig 1).

Archaeological evidence directly supports that the San did find and transport fossils over long distances [7,8], and could interpret them in surprisingly accurate ways [11,12]. If the San could identify the fossilised skulls of dicynodonts as belonging to once alive animals, it is possible that their tusked faces could have contributed to their rock art. In this respect, it is noteworthy that, to the San of the Koesberg, the animals depicted on the Horned Serpent panel were real and used to live among them: “The Bushmen of the east […] declare that there were at one time a number of animals living in the country in the days of their forefathers, which are now extinct and nowhere to be found in Southern Africa. Some of these are described as great monstrous brutes, exceeding the elephant or hippopotamus in bulk.” (p. 131 in [14]). In the next page of the same book, the tusked animal is described as an entity distinct from the rain-animal (referred to as ‘Kou-teign-Koo-rou) and the Serpent (referred to as ‘Koo-be-eng) [14]. In addition to its tusks, the extraordinary size of the animal evokes the heavily mineralised bones and disproportionately enlarged skulls of some dicynodonts found in abundance in the Main Karoo Basin, such as *Kannemeyeria*, *Lystrosaurus*, and *Daptocephalus* [2,6,28,30]

If the La Belle France painting pictures a dicynodont, it would have been made independently from, and perhaps before, the discovery of *Dicynodon lacerticeps*, the first dicynodont described in the western scientific literature [31]. This could be the oldest depiction of a dicynodont ever made. This possibility is intriguing and warrants scrutiny.

In this contribution, the Horned Serpent panel was re-examined to address its accuracy and determine its age. The accuracy of Stow and Bleek’s [13] reproductions has been questioned, which may alter significantly the interpretation of the depicted scenes [32], and as such, first hand photographs of the paintings were made. The palaeontology and geology of La Belle France were also explored to assess the fossil richness of the area and its potential for supporting a geomyth.

## Material and methods

**Fig 2:**
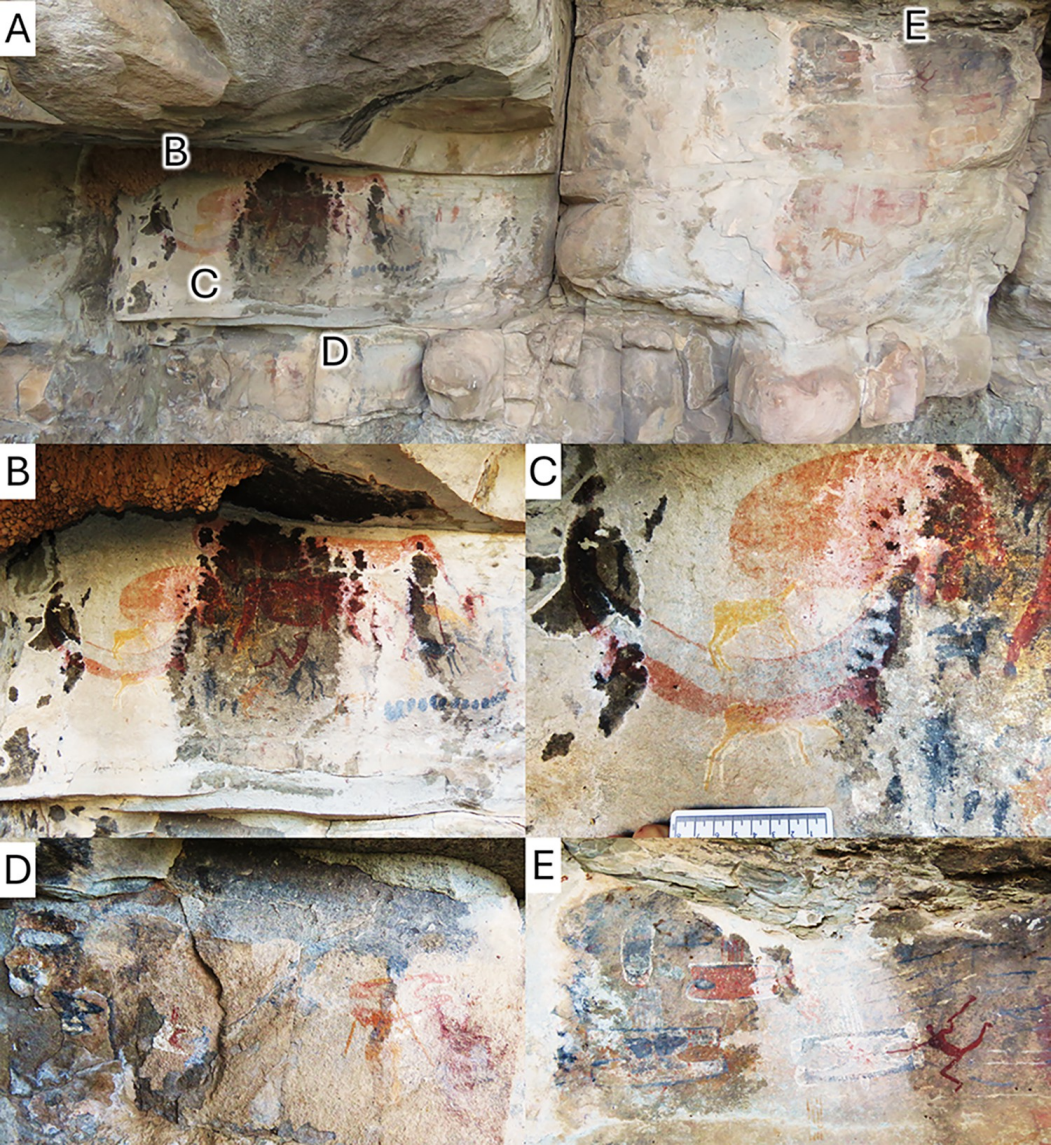
The Horned Serpent panel. A, general view of the Horned Serpent panel photographed in 2024 by the author. B, close up of the section figured in Stow and Bleek’s [13] plate 39. C, close up of the tusked animal. D, close up of the warriors painted below the Horned Serpent panel. E, close up of the warriors painted to the right of the panel.

**Fig 3:**
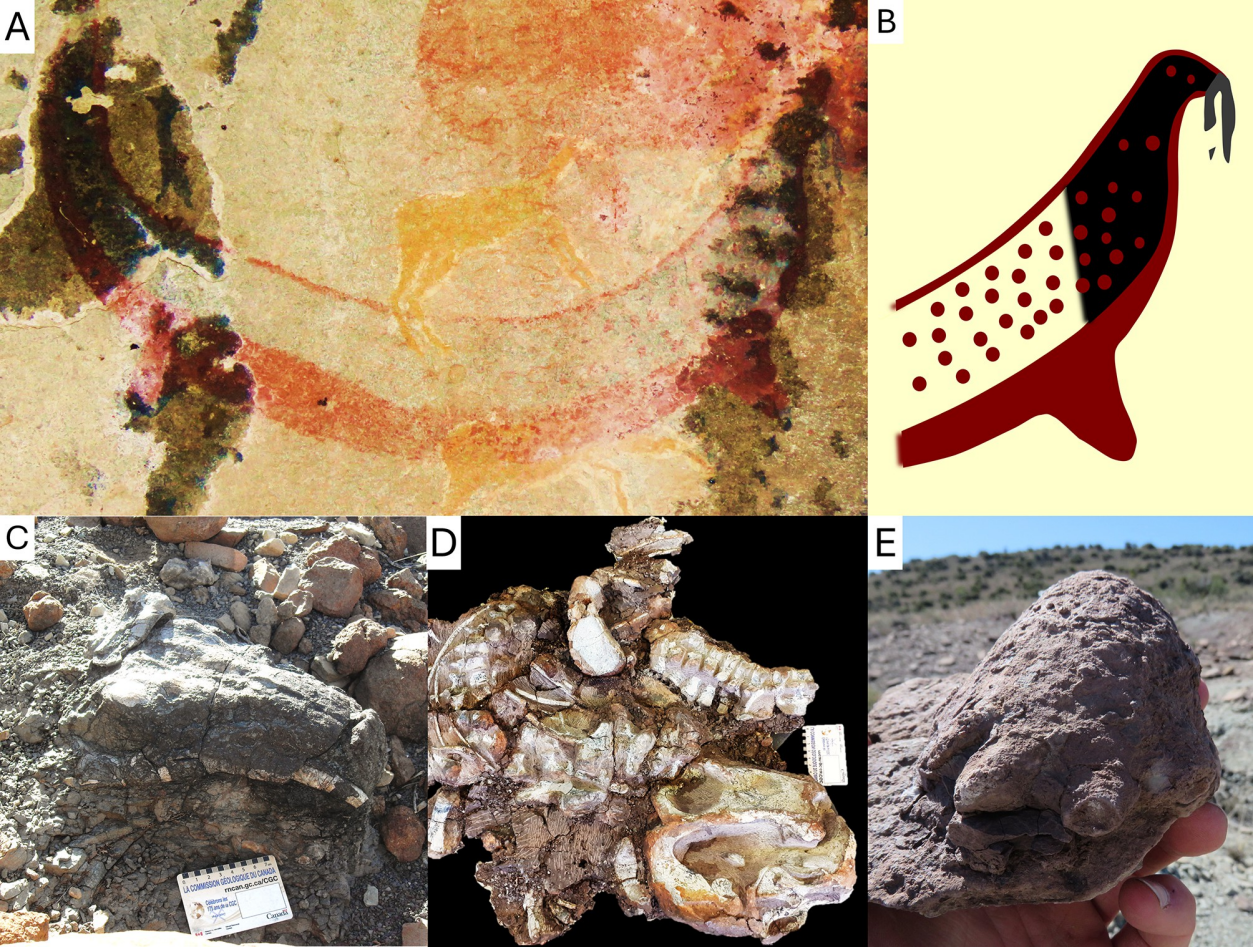
Interpretation of the tusked animal of the Horned Serpent panel and its dicynodont-like traits. A, Photo of the tusked animal of the Horned Serpent panel. B, interpretive drawing of its head. C, skull of a *Lystrosaurus* (14-03-2024, Oviston Nature Reserve) showing the prominent tusks, photographed in situ at the moment of its discovery, before excavation, and unprepared. D, complete skeleton of a *Lystrosaurus* (BP/1/9100, Oviston Nature Reserve) with its vertebral column curved into an opisthotonic "death pose", ex situ, prepared. E, the ‘mummified’ foot of a *Lystrosaurus* (28-08-2022, Fairydale, Bethulie District) showing the warty aspect of its preserved skin, ex situ, unprepared.

The paintings were located thanks to the help of Mr. and Mrs. Swanepoel, the landowners, and Mr. and Mrs. Kruger, to whom the author is infinitely grateful. The paintings were photographed using a regular camera. Fig 2A shows what the photographs look like without enhancement, but the closeups in Figs 2B–2E and 3A are slightly enhanced for readability (contrast, temperature, and saturation).

Stow and Bleek [13] locate the “Brakfontein Cave” on farm La Belle France (plot La Belle France 458), which is misleading because the paintings are neither on La Belle France 458 nor Brakfontein 83, but on plot Alpha 872. All three farm plots belong to the same landowner and access to Alpha 872 is through La Belle France 458, so to avoid confusion and ensure continuity with Stow and Bleek [13], the locality will be referred to simply as La Belle France throughout this work.

La Belle France was prospected by the author for one day to address the presence of fossils in the immediate vicinity of the paintings. Fossils were photographed in situ, positioned using a handheld GPS, and consolidated with Paraloid B72 glue. Given their possible association with the nearby rock art, the fossils were not collected. No permits were required for the described study, which complied with all relevant regulations.

Abbreviation: BP, Evolutionary Studies Institute of the University of the Witwatersrand, Johannesburg, South Africa.

## Results

### Paintings

The Horned Serpent panel is located at GPS coordinates S30°15.776’ E26°54.002’. The panel is well preserved (Fig 2A–2C), except for the drawings made with black pigment, but still depicts most of the elements redrawn by Stow and Bleek [13]. The locality is classically known as the “Brakfontein Cave”, but it is rather a rock shelter under a small sandstone overhang. This overhang and the site are located on the western side of a north-south ridge, a few kilometres west of the main Koesberg mountains. The paintings are made on a five-metres-thick exposed section of the Molteno sandstone (see the Geology and Palaeontology section below).

The complete panel is about 1.20 m long (Fig 2A) and is part of a larger mural that is about 10 m long (see The African Rock Art Digital Archive: http://www.sarada.co.za). Consequently, Stow and Bleek [13] plates 36 to 39 illustrate only a small fraction of the paintings available at the site. These include, but are not limited to, unpublished paintings of a feline, aardvark, many eland and other antelope, and hunting and battle scenes, description and interpretation of which are beyond the scope of the current work.

The tusked animal itself has a slightly longer and more slender body than in Stow and Bleek’s [13] reproduction (Figs 1 and 3A). The presence of tusk-like structures is verified. They are not an artifact of Stow and Bleek’s [13] reproduction, and they do not appear to belong to another element of the painting. Stow and Bleek [13] figured the tusk-like structures as being the same red colour as the rest of the body (Fig 1), whereas tusks are usually depicted in a different, lighter colour in most San rock art [19–21]. This could have weakened the interpretation that they may indeed be tusks; however, the new observations made here show that the tusks were painted in a different, more greyish colour than the black head of the animal (Fig 3A and 3B). This conforms to the usual depiction of tusks in San art.

Alternatively, these tusk-like structures may be interpreted as a fish or a snake being held in the mouth of a larger animal (such as a seal). Snakes are sometimes depicted as if they were held in the mouth of another animal (see plate 67A in [13]), but the inverted U shape formed by the tusk-like structures is far too short and featureless to conform to the usual depiction of snakes in San rock art [13,21–25]. The same applies to possible fish and eels. Another possibility is that the tusked animal could, in fact, be bleeding from its nose rather than sporting tusks, which is quite common in the San rock art [33,34]. This can also be safely excluded as nose-bleed are painted in red and usually displayed as exaggeratedly long and slender sprays in San rock art [25,33–35]. The tusk-like structures are finally too thick to be whiskers or rain-spitting [25]. Overall, they best match downward pointing teeth, consistent with dicynodont tusks (Fig 3A–3C). Their irregular shape compared to real tusks may be due to damage combined with the coarse grain and irregular surface of the sandstone, which made the drawing of this small element difficult.

Finally, the new observations show that the Horned Serpent panel is integrated in a larger scene that includes Sotho/Tswana and IsiZulu warriors, identifiable by their shields (Fig 2A, 2D and 2E). The San depiction of IsiZulu warriors battling with the Sotho/Tswana suggests that they were not made before the Mfecane, circa 1821–1828 [14,22]. As such, assuming that the warriors were painted at the same time as the tusked animal, the rock art at La Belle France may not be older than 1821. Most of the San retreated to the Maloti in 1835 and had completely abandoned the area by the 1850s [14,36,37], which gives the youngest possible age of the paintings. Consequently, the tusked animal was likely painted during this short interval, between 203 and 189 years ago, but could be older. Some paintings in the area have been dated to between 300 to 4000 years old [38–40], but previous records of the Horned Serpent panel (see The African Rock Art Digital Archive: http://www.sarada.co.za) demonstrate that the rock art at La Belle France is fading rapidly (especially the black pigment), so a younger age of about 200 years old is favoured here.

### Geology and palaeontology

**Fig 4:**
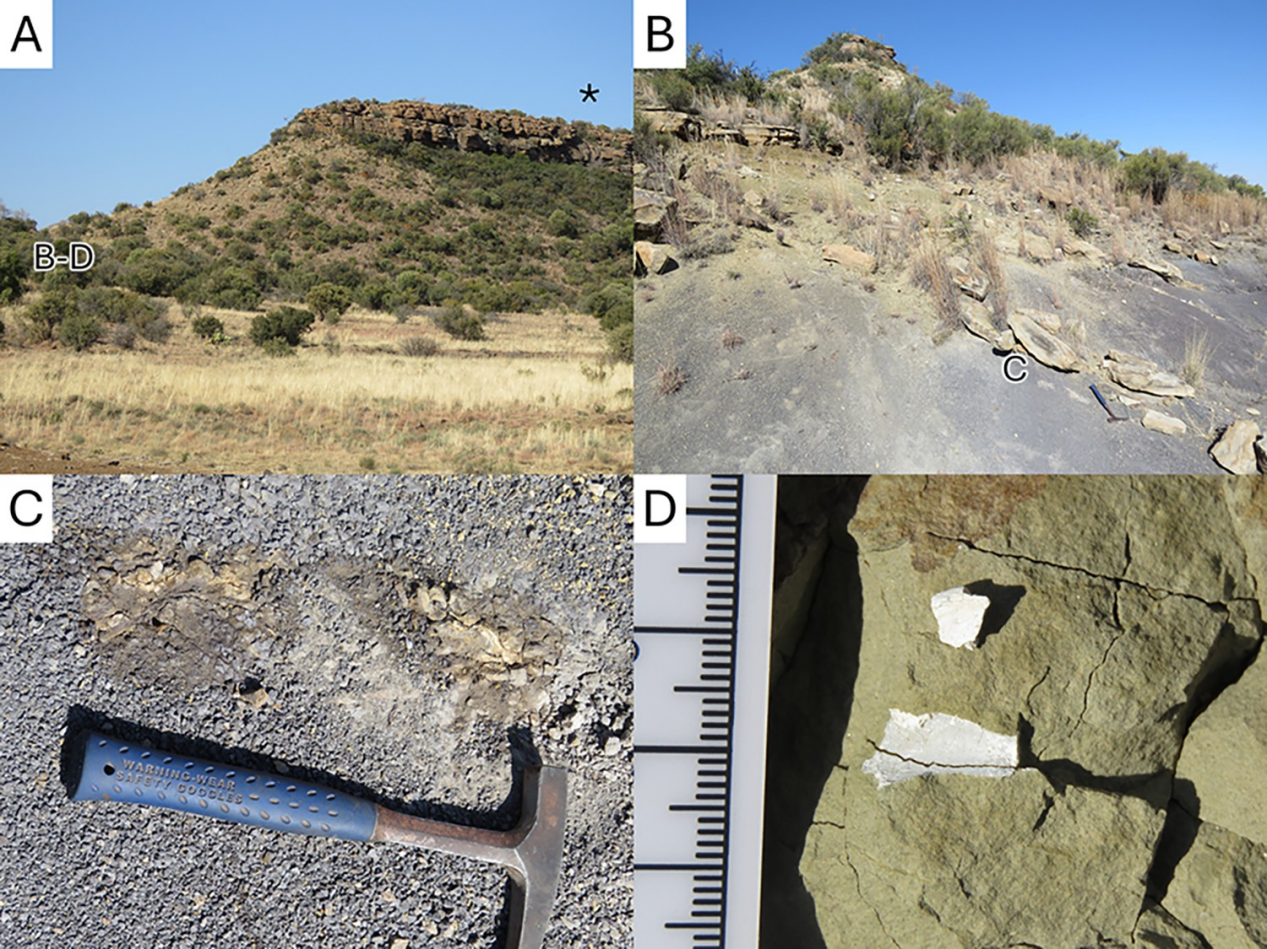
Pictures of the La Belle France locality. A, general view of the outcrops, from the west. The paintings are located below the * and the fossils were found where marked B-D. B, a siltstone outcrop. The position of the fossil in panel C is marked by a small C on the picture. C and D, close up views of some of the in-situ bone fragments discovered.

The La Belle France paintings are located on the western face of a north-south-running dolerite dike, west of which the Molteno Formation is exposed (Fig 4A). The exposures preserve at least three cycles of alternating conglomerate, sandstone and blue to green siltstone that likely represent the Indwe Sandstone Member of the Molteno Formation [41]. The paintings were made on the five-meter-thick section of the uppermost layer of sandstone, at the top of the ridge. Fossil bones are rare in the Molteno Formation [1,41], but, in the course of this work, abundant bone fragments were found in ex situ blocks of conglomerate. More importantly, in situ bone fragments were found in a gulley exposing blue to grey-green siltstone outcrops, some 50 m north of the paintings, at GPS coordinates S30°15.670’ E26°53.960’, S30°15.660’ 26°53.951’, and S30°15596’ E26°53.997’ (Fig 4B–4D). They are not identifiable beyond tetrapod indet.

## Discussion

The La Belle France animal is unique in San rock art. Its back is curved as a wide U, which is often found in paintings of fish and half-fish therianthropes [13,42,43]. This connection to water, coupled with its spotted skin and association with a horned snake are all consistent with its interpretation as a rain-animal [25,43]. As stated above, rain-animals are fantastic in nature, but they were arguably inspired by real-life elements [23,42].

The general outline of the La Belle France animal is evocative of a skink (such as *Mochlus* spp.). It has a small and rounded head, thick neck, long trunk and tail, short limbs, and a dark and spotted skin [44]; however, skinks are small, fossorial, and easily scared animals, and the ethnographic literature indicates they did not leave much impact on the San culture (unlike snakes and agamids, for instance; [13,14,26,45]). Moreover, unlike the La Belle France animal, lizards are always depicted in dorsal view in South African rock art, with all four legs visible, and the rare counterexamples are limited to therianthropes only [13,22,45,46]. So even though, at first glance, the body proportions may be consistent with skinks, this is not a parsimonious interpretation considering their inconspicuous nature and the ethnographic literature.

The animal depicted on the Horned Serpent panel bears two prominent structures, here best identified as tusks. They resemble those of the fossilised skull of a dicynodont (Fig 3A and 3B), consistent with the local San myth that designates this animal as an extinct species [14]. Its tusks point straight down, unlike in the San’s depictions of other tusked animals (real or fantastic), in which the tusks always point upwards [19–22]. It also differs from an elephant by the absence of a trunk. Carnivorans, rock hyraxes, and hippopotami are usually depicted with their mouth closed in San rock art, but when they do show their teeth, both the upper and lower canines or tusks are clearly depicted [21–25]. The tusked animal of La Belle France has upper tusks only, like a dicynodont (Fig 3A–3C). It, thus, does not appear to be a living animal, or a creation based partly on an existing animal.

Moreover, the tusked animal assumes a pose with its back curved as a wide U (Fig 3A), which is unusual in San rock art (except for fish [13]). This is evocative of the opisthotonic “death pose” often displayed by fossilised skeletons in the area (Fig 3D) [47]. The skin of the tusked animal is dotted (Fig 3A and 3B), which is not unusual in San rock art, but is also consistent with the warty mummified skin preserved in some dicynodont fossils in the area (Fig 3E) [5]. The depiction of short limbs on the panel (Fig 3A) may be because, in the Karoo Basin, the limbs are often buried deeper than the rest of the body (if the animal sunk into the mud) or are the first to disarticulate from the rest of the body before fossilisation [5,47,48]. The discovery of partly buried or disarticulated skeletons coupled with the intrinsic spiritual nature of these long dead “great monstrous brutes” (as related in Stow and Theal [14]) may be responsible for the liberty taken in picturing the body proportions of the tusked animal. For instance, rain-animals are often pictured as aquatic beings [23–25], which may also account for the seal-like body of the tusked animal. All these elements are consistent with the hypothesis that the tusked animal may have been inspired, in part, by the discovery of dicynodont fossils.

**Fig 5:**
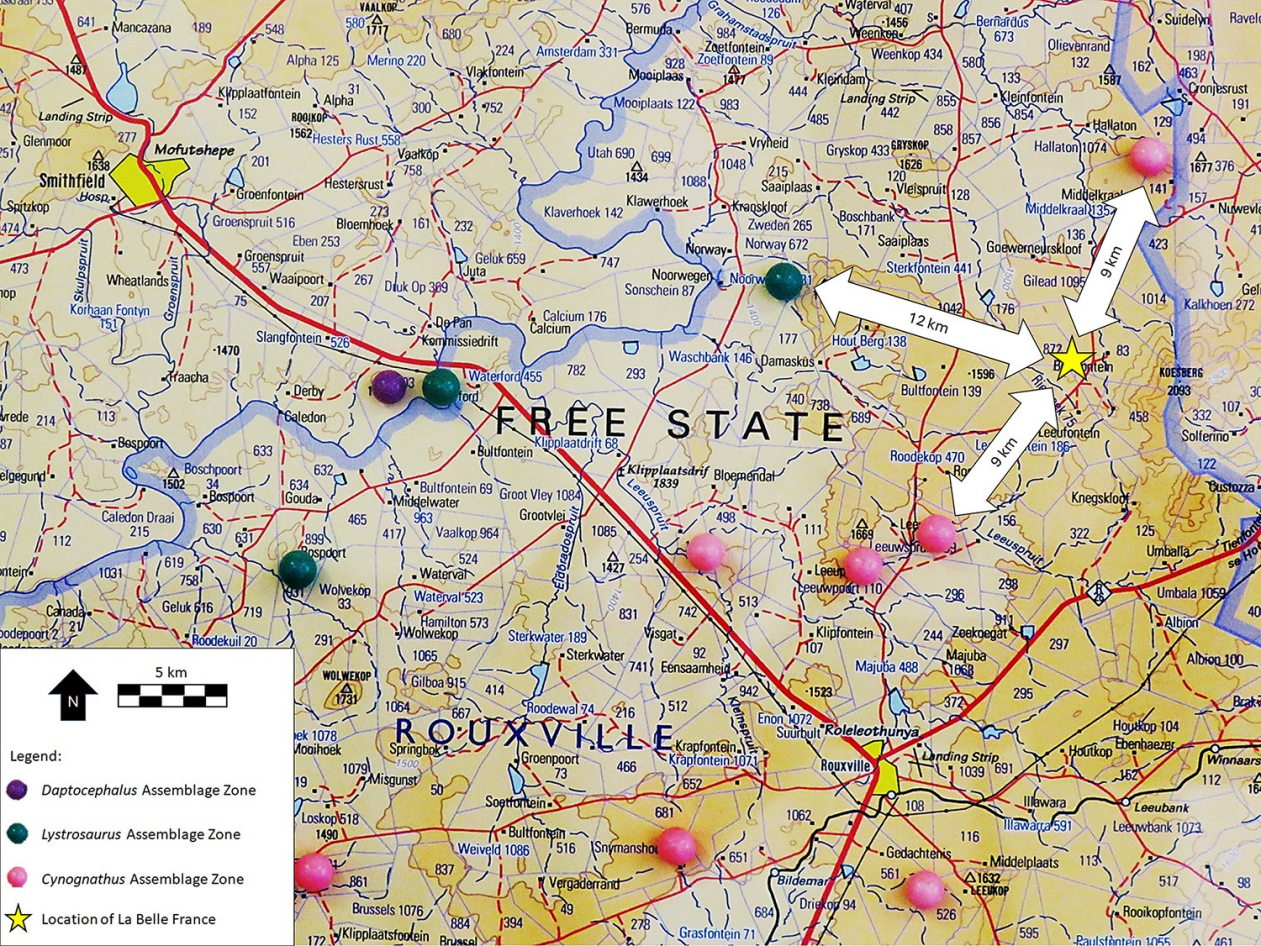
Excerpt of James Kitching’s wall-sized compilation of 1:250 000 topocadastral maps made in 1972. The position of La Belle France is marked with a star. Outcrops are marked with coloured pins. The colours correspond to the Assemblage Zones as identified by Kitching [28] based on the fossil fauna.

As stated above, tetrapod fossil fragments are not rare in the immediate vicinity of the paintings (Fig 4C and 4D); but more importantly, La Belle France is located immediately near Rouxville and 60 km from Aliwal North, in an area where the Burgersdorp Formation of the Beaufort Group is well exposed [28,49,50]. This formation encompasses the *Trirachodon-Kannemeyeria* Subzone of the *Cynognathus* Assemblage Zone, which is famous for producing countless bones belonging to the dicynodont *Kannemeyeria* [49], a bull-sized animal which would match the description provided by the San of a “great monstrous brutes” [14]. The closest outcrops of the *Cynognathus* Assemblage Zone are located 12 km away (Fig 5). Other rich fossiliferous areas nearby include those where the underlying Katberg Formation of the Beaufort Group is exposed, and in which the dicynodont *Lystrosaurus* is found in great numbers [28,47,51]. The closest outcrops of the corresponding *Lystrosaurus* Assemblage Zone are less than 10 km from La Belle France (Fig 5), towards Smithfield [28,50]. One of the richest localities, Fairydale (Donald 207, Bethulie District) where hundreds of dicynodont bones and skulls were found, is located just a 100 km away [5,29]. The occurrence of stone tools alongside the fossils demonstrates the occupation of these palaeontological sites by the San, as has been documented since the pioneering work of D. R. Kannemeyer and A. Brown [52–54].

The ability of the San to observe and interpret their environment in an essentially scientific way is well-known [55–57]. Their tracking skills are famous for the accuracy of the data they can extract from footprints [27,55–57]. They also use the same names across different species to designate muscles and bones, which implies that they understand the notion of homology [27]. It is well-established that the San from the Karoo were aware of the geological items and fossils in their surroundings, as they sometimes collected and made paintings of the fossils they found [7,8,11]. The possible depictions of dinosaur footprints at Boesmankoppie (Northern Cape Province, South Africa) and the Mokhali Cave (Lesotho) exemplify how the San could integrate fossils into their rock art [11,12]. At Schaapplaats and Avondzon (Free State Province, South Africa), the paintings do not depict any fossil or extinct species, but the painted surfaces are so close to conspicuous dinosaur footprints and bones, respectively, that the artists most likely noticed them [7]. Fossil awareness among the San was not limited to footprints, as the manuported dinosaur bone of Bolahla (Lesotho), dated between the 12^th^ and 18^th^ century, demonstrates [58]. Ellenberger et al. [11] also noted the presence of a dinosaur skeleton on the path to the Mokhali Cave, 10 m from the site, that would have been noticed by the San. These examples are mostly concentrated in the Stormberg Group (in the Drakensberg), likely because rock art and archaeological sites are better preserved in the rock shelters and caves of the Clarens Formation, but they do suggest that the San were capable of finding fossils, including bones. As dicynodonts are very abundant in the underlying Beaufort Group but exceedingly rare in the Stormberg Group [28,30,59], the tusked animal on the Horned Serpent panel could be the first evidence of San palaeontology in the Beaufort part of the Karoo Basin.

## Conclusion

The ethnographic, archaeological, and palaeontological evidence are consistent with the hypothesis that the Horned Serpent panel could possibly depict a dicynodont. This is supported by i) the downward orientation of the tusks on the La Belle France tusked animal (Fig 3A and 3B), which does not match that of any modern African animals, but does match the tusks of dicynodonts (Figs 1B and 3C), ii) the abundance of dicynodont fossils in the area (Fig 5), and iii) the local San belief into a long extinct, large animal [14]. This would imply that the San may have i) discovered dicynodont fossils, ii) interpreted them as long-extinct species, iii) made a painting of one of them at La Belle France, and iv) integrated them into their worldview. If so, this would evidence that Later Stone Age people were aware of dicynodont fossils at least a decade before their formal scientific description by western scientists [31], and made the first known reconstruction of one of them. Even if one considers that the Horned Serpent panel has a purely spiritual meaning, it does not invalidate the hypothesis that the tusked animal itself may have been imagined based on a dicynodont fossil. The spiritual and palaeontological interpretation of this painting are not mutually exclusive. Helm et al. [7] were the first to propose that some of the strangest animals in the San rock art may be interpreted as depictions of Permian reptiles, and the current contribution supports that perhaps there may be more dicynodont depictions to be found among some of the paintings and petroglyphs preserved in the Karoo [7,19–21,60,61].
